# Perceptions and attitudes of Nursing students towards end-of-life care: a Phenomenological Study at a tertiary hospital in Uganda

**DOI:** 10.21203/rs.3.rs-4594723/v1

**Published:** 2024-07-23

**Authors:** Asha K. Nabirye, Ian G. Munabi, Aloysius G. Mubuuke, Sarah Kiguli

**Affiliations:** Makerere University College of Health Sciences

**Keywords:** End of Life care (EOLC), Caring for dying patients, Nursing students, Perceptions, attitudes

## Abstract

**Introduction:**

Clinical practice is an important part of nursing education as it helps nursing students transition into competent health professionals who can provide quality services. However, research studies convey it as a significant stressor for nursing students when they are obliged to end of life during clinical practice. The perceptions of nursing students on caring for end-of-life care have not been exhaustively studied in the Ugandan context. This study was conducted to understand nursing students’ perceptions of end-of-life care during clinical practice at Mulago Hospital.

**Methods:**

It was a qualitative study with a phenomenology method carried out among 15 nursing students from Makerere University who were assigned to Mulago Hospital for clinical practice. Data was gathered using an in-depth interview guide, audio was captured, and transcriptions were analyzed thematically using Atlas.ti version 6.

**Results:**

Three main themes emerged from the data; two themes on perceptions were (i) reactions when nursing students were informed about the physical process of caring for dying patients and (ii) how student ideas about the care changed during the process of caring for end-of-life. The third theme was the attitude of students towards patients when offering end of life care. participants described their reactions as chaotic, devastated and scared about their first-time encounter with caring for dying patients, the physical process also required preparation to handle the situation and understanding, and for positive personal values felt respected, appreciated, trusted and valued when informed about the physical process of caring for the end-of-life

**Conclusion:**

In this study, nursing students held divisive attitudes on care for dying patients. However, nursing students appear to transition from unfavorable impressions of the process and encounter of caring for dying patients to positive perceptions during the actual caregiving experience. Despite their differing perspectives on caring for dying patients, students were typically empathic towards them.

## Background

Caring for end-of-life is one of the serious concerns of the healthcare systems and settings [1] globally due to the increased prevalence of chronic conditions, the number of patients dying and in need of end-of-life care (EOLC) [2]. Nursing staff and student nurses are the most consistent providers of care to end-of-life [3, 4]. The nurses provide care at health facilities or homes to help dying patients cope with physical limitations and protect their dignity. The purpose of caring for end-of-life is not to prolong life but to control pain and other symptoms so that the quality of life is increased to experience an honorable and peaceful death [5].

Caring for end-of-life is comprehensive and bridges the communication gap between patients, families, and healthcare team members which is a key role of the nurses[6] because the nurses’ knowledge of the patient’s wishes is used to support the patient and provide information to other members of the healthcare team[7]. The attitudes and perceptions held by nurses attending to end-of-life significantly impact the quality of care provided [8].

Despite the need for specialized education to prepare nurses for the profound experiences involving death and dying, only a few nurses attain it[9] and student nurses receive limited exposure and preparation for end-of-life care [9, 10]. Nursing students need to comprehend how to handle a patient’s death, providing compassionate support to the patient’s family, friends, and loved ones as they grapple with death and psychological trauma [11].

Failure to provide appropriate care affects the dying patients negatively because of their increased risk of adverse events related to multiple biological factors, use of invasive medical devices and treatments, and vulnerability to social isolation and family abandonment[12].

In Uganda, there is a Limited understanding of nursing students’ sentiments towards caring for end-of-life yet it is pivotal in nursing education to prevent late referrals, poor integration of dying patients to end-of-life care by nurses, and poor health service provision. Most of the available literature on this subject is from studies done in developed countries and there is hardly information specific to the Ugandan context. The need for nursing students and future nurses to possess the knowledge and self-awareness necessary to assist individuals through these frequently distressing situations is paramount as nurses often remain present throughout the dying process to support both the patient and their family. Additionally, there is limited access [13] and provision of care to dying patients [14] because of the existing inequalities in the socio-economic status, limited number of trained health teams, knowledge, and skills, and frequent shortages of essential medications. Nurses use their experiences to evaluate and diagnose problems and validate the patient’s perceptions hence reducing the burden on caregivers[15]. Nurses are responsible for caring for dying patients but are challenged with unique individual interpretations and perceptions about the death experience [16]The primary research question of this study was how do student nurses perceive providing end-of-life care to dying patients during clinical placement?” This study aimed to document the perceptions and attitudes of nursing students caring for end-of-life in the Ugandan context. This is necessary to provide data to foster focused training and palliative care simulations which help students shift their attitudes and beliefs and improve the care they give to patients in need of at end-of-life care services in Uganda.

## Materials and Methods

### Study aim

This study aimed to provide an in-depth exploration of nursing students’ perceptions on caring for dying patients during clinical practice at Mulago Hospital.

### Study design

A qualitative study employing a phenomenology approach was carried out to understand the perceptions of nursing students on caring for dying patients.

### Study site

The study was carried out at Mulago hospital which is a national referral hospital and teaching facility of Makerere University Collage of health Sciences. The hospital has 1790 beds with specialized services admitting over 140,000 patients and with 600,000 out patients annually. This site was chosen because it’s the national teaching hospital with large numbers of patients and it is where students of Makerere University are usually positioned during their training and clinical practice

### Selection of participants

The Participants were purposively selected and interviewed until the data saturation point was attained on the fifteenth student. The study sample consisted of fifteen undergraduate Nursing students of year III and year IV. They were nine males and six female students of Makerere University who had been for clinical placement, cared for dying patients in the last six months prior to data collection, and consented to the study. The study did not include nursing students who were not physically available at the time of data collection and those that who were ill to participate in the study.

### Data collection

The interview guide was developed for this study (Refer to appendix A). In-depth interviews were conducted from the 14th of June 2023 to the 24th of June 2023 among students who had been on ward for placement, were eager and consented to take part in the study. Audio-recorded interviews were conducted in English language, by two registered nurses with experience in data collection, and each lasted between 25 and 60 minutes.

### Data management and analysis

The audio-recorded interviews were verbatim transcribed and transcripts were exported to Atlas. ti version 6 software for analysis. Inductive analysis was employed in a thematic analysis. The three procedures outlined by [17] were used for data analysis. The first step was a cursory read-through of each text to get a feel for it and generate concepts for more in-depth research. The second phase was structural analysis, which exposed the structure and internal dependent relationships that make up the static state of the text and helped to find important assertions. Understanding the interpreted whole through reflection on the superficial reading and structural analysis was the third phase.

Internalization of transcripts was done through superficial reading, thematic indexing, and structural analysis. The key phrases that directly addressed how people perceive caring for dying patients were extracted from each transcript. The significant statements were translated into meanings. The formulated meanings were then organized into themes, allowing for the emergence of topics that were present in the transcripts of all the participants. The outcome was then included in a thorough, in-depth account of the phenomena.

Each transcript was opened in the Atlas. ti 6 program, and each statement was read and coded line by line. The coding was examined, with some codes being combined and others being modified to create themes. The development of a codebook was followed by the identification of main and sub themes. Also, the Compare and contrast method was used to verify the findings.

### Ethical considerations

The study was approved by the Makerere University School of Medicine Research Ethics Committee (SOMREC) under REF MAK-SOMREC-2023-581. The participants gave written informed consent to participate in the study. The lead researcher informed the nursing department chair at Makerere University and got written approval from everyone who took part in the study. The information that respondents provided was treated as confidential information, and it was utilized such that it was impossible to link a specific response to it. The research team treated respondents with respect, particularly respecting their opinions, including their right to end an interview when they felt uncomfortable.

## Results

The results presented in this section were from fifteen Key informant interviews conducted among fifteen nursing students in the third and fourth year of medical school between the 14th and 24th of June 2023 and, had cared for the dying patients at Mulago Hospital. There were nine males and six females.

**Table:** 

Theme	Subtheme	Codes
Theme1: Being informed about the physical process of end-of-life care	1.1Reactions when informed about physical process	1.1.1: Chaotic, eager, expectant, devastated, scared
	1.2 Understanding of the physical process	1.2.1: Requires preparation to handle situations & knowledge 1.2.2: Thoughts about the task: Hard for the first-time students: not a nice experience 1.2.3: Students thoughts on relevance of the task: Biased and no need to care for dying patients.
	1.3 Personal values attached to understanding the physical process	1.3.1: positive Personal values: respected, appreciated, trusted, valued. 1.3.2: Negative personal values are: not trusted by family members of patient, un worthy to care for other improving patients, devalued, not easily acceptable and confused
Theme2: change in ideas	2.1 Survival of patient	2.1.1 Students thoughts about survival of patients: chance, someone can even survive at last moment and optimistic 2.1.2. Student’s thoughts & beliefs about dying patients changed: they are not bad people but deserve care, dying patients don’t hurt people and caring for them will not kill other people. 2.1.3. Student’s thoughts about altering the situation/process: they always think something can be done, acknowledgement of death as a fact and reality, un avoidable.
	2.2 Need for care	2.2.1. Students thoughts before clinical placement: the dying patients don’t need care, they are going to die anyway, wastage of efforts therefore no need to care for them. 2.2.2. Students thoughts after clinical placement are: dying patients have a right to be cared for, dying patients deserve less painful death and dying patients deserve a peaceful death. 2.2.3. Social responsibility: caring for patient consoles family members and consoles health professionals
	2.3 Importance of the clinical placement	2.3.1. Importance of the clinical placement: lessons learnt, built/gained confidence to care for similar patients, applied lessons learnt, always do the best, improved nursing skills, experience worth it for the knowledge, worth the learning experience, more confidence after continuous exposure, not feeling so nervous, learning opportunity, opportunity to gain experience, offer holistic care, practice theory from class,
Theme3: Students' attitudes to dying patients	3.1 Empathetic Resourceful Persons	3.1.1. Continue to offer care, best efforts applied, obligation to offer a painless death, relieve patient pain, responsible for care for relatives' sake, interested to overcome productivity shame (guilt feelings if not care) 3.1.2 provide moral support: Keep positive talk with patients, reassure them, comfort them,
	3.2 Informants in Clinical practice	3.2.1 source of comfort to patients, Source of information to patients, only available support, address loneliness of patients (some patients have no one to talk) 3.2.2. uncomfortable to perform some clinical procedures, when overwhelmed by patient’s condition, prefer to avoid physical presence
	3.3. Sympathetic & protective of relative's reactions & feelings	Code:3.3.1 protective of relative's feelings by continuity of care, considerate in communication, assumes effects to family (e.g loss of person, bread winner, family breakage, wasted family efforts) 3.3.2. provides counseling, reassurance and comfort, kept hopeful
	3.4. Cooperate with relatives	3.4.1: informed about physical process, involved in physical care e.g. feeding patient, 3.4.2: relatives are appreciation of care given

The nursing students’ statements on end-of-life care were grouped under three themes which were drawn from many codes. The themes that emerged were (1) reactions when nursing students were informed about the physical process of offering end-of-life care (2) how their ideas about the care changed during the process of end-of-lifecare and (3) Student attitudes during end-of-life care.

### Theme1: Being informed about the physical process of end-of-life care

This theme comprised of three sub themes in regards to the reactions, understanding and personal values attached when nursing students were informed about the physical process of end-of-life care as described below:

### Subtheme 1.1 Reactions when informed about the physical process of offering end-of-life care

The participants described their reactions as chaotic, devastating, and scared for the first encounters when they were informed about caring for dying patients, which was negatively perceived. However, some students described positive reactions including eagerness and expectancy to be part of the medical team.

…. chaotic… So, to me, I was like, now how am I going to do this to people who are already even giving up to this scenario, which is coming ahead of them? How am I going to be able to explain to them it was like, also, even the patient was at that stage where he saw that nothing can be done any further to improve…. I thought I was not ready yet to do that. Because I felt I needed more time, you know so that I first watch people do it. And then with the time, I also first do it with assistance, and then at least maybe they can give me but then in my clinical rotation, it was I think, my first week there, and they just told me go and care for the dying patient. And that alone triggered a lot of things in my mind. Oh, yeah. … (Student Nurse Participant 10)

### Sub theme1.2: Students’ Understanding of the Physical Process

The participants had an understanding that the physical process required preparation to handle the situation and knowledge, however, during this clinical placement; they expressed that they were not adequately prepared, were rushed, and were not sure of what to do during the physical process of care for dying patients. The participants also thought it was a hard torturous experience and undermined the relevance of care to the dying patients.

“…And even like being my first time, first of all, figuring out what to do at that point, became a little hard for me……. I felt like since someone’s dying, I felt at some point like, my, my efforts were going to be a little wasted… (Student Nurse Participant 1)’.

### Sub theme1.3: Nurses attached Personal values to understanding the physical process

The participants’ understanding of the physical process was also expressed in their mixed views of personal values. For instance; some participants with positive personal values felt respected, appreciated, trusted, and valued when informed about the physical process of caring for the dying patients. Some participants viewed this call as though they were not trusted by fellow health workers, not trusted by family members of the patient, unworthy to care for other improving patients, devalued, not easily acceptable, and confused.

…someone has appreciated I value that, yes, I can only participate in this. So, I also feel my presence and the word that yes, if I’m called upon to participate in this caring for this patient who is actually going to lose his life. I felt valued and trusted… (Student Nurse participant 4)

‘…I felt like they don’t have enough trust in me that I would do something with a patient… (Student nurse participant 3).

### Theme 2. Change in ideas before and after caring for dying patients

This theme relates to the various changes in views of students regarding caring for dying patients. That included sentiments on survival, need for care and importance of clinical placement.

### Sub-theme 2.1 Sentiments on Survival of Patient

The participants revealed changes in thoughts about the chances and possibilities of survival of dying patients even though this was not the case before the clinical placement. Some participants who always thought there was something always to be done to alter the dying process got to acknowledge the fact and reality of death as inevitable in some situations.

The participants’ Childhood thoughts and beliefs about dying patients also changed and were no longer adaptive to them; such as beliefs that dying patients are bad and harm caretakers. After this clinical placement, the participants changed their view on this and understood that dying patients are not bad people but deserve care, they don’t hurt people and caring for them will not kill other people.

‘…. I’ve never believed that somebody can really die when I’m seeing and really, they are dead, you know? So, for me, I thought maybe they were just sleeping. And maybe they would wake up sometime. I never cared much about dying patients. But after this experience, I learned that patients actually die……. (Student nurse participant 10)”.

### Sub-theme 2.2 Sentiments on Need for End-of-life Care

In their view of significance of dying patients, participants before clinical placement thought this care was not necessary and that dying patients don’t need care after all they are going to die anyway. They were biased and thought the physical process of care is a waste of effort. However; after the clinical placement, their ideas on the need for this care changed, and realized dying patients have a right to be cared for, and they deserve less painful and peaceful deaths. Hence, they began to handle the physical process of care as an obligation to relieve patients of pain as an offer of painless death.

… Initially, I believed that they were wasting time. Because the first patient I was assigned to care for died or was in the process of dying, they did not inform me. So, they just informed me when to modify the oxygen for that individual, when to give this medication, when to provide that, and then later, I had them discuss how the patient is dying and whether we should refer them to palliative care. Yes... And I was thinking, if you already know this guy is dying, why are you sending me there again to provide medication and so on? It’s probably best if we halt everything now that he’s already dying. I’ve never been very concerned about dying patients. But afterwards (Student Nurse Participant 7).

The participants also regarded the physical process of care after clinical placement as social responsibility, for instance; it provides consolation to family members and health professionals.

‘……. There, you realize that everyone has value and is valued by their family members and so these people have a right to experience or to be treated better in their last days…. (Student nurse participant 3).

…it causes breaks. Like many families, the progress of many families comes down, if really, the person that I’ve lost has been key has been the one standing…. (Student Nurse participant1).

### Sub-theme 2.3. Sentiments on Importance of the clinical Placement

The participants also expressed their change in thoughts on the importance of the clinical placement, from torturous to realizing that was a learning lesson, which built their confidence to care for similar patients. They acknowledged that the placement improved their nursing skills, and knowledge, and was an opportunity to offer holistic care and obtain great experience for the application of medical care.

……. I never cared much about dying patients. But after this experience, I learned that actually dying patients need to be cared for. …. (Nurse Participant 10)”

…I was just hearing about central lines, but I had actually never cared for a patient with a central line…it was my first encounter (nurse participant 8)

‘…. I kept on first of all, learning a lot. I learned a lot. And I kept also, interacting with my fellow students who were with me in their own… (Nurse participant 1).

### Theme 3: Students’ attitudes to dying patients

This theme relates to the student approaches and positions toward end-of-life care including: creating empathetic relationship, informants in clinical practice, sympathetic to relatives and cooperate with relatives as expressed below:

### Sub theme 3.1: Create Empathetic Relationships with Patients

The participants continued to offer care as they viewed themselves as resourceful persons, who were driven by empathy to relieve the patient of the great pain, and valued the responsibility of care for the sake of relatives. More to that, their empathy was driven by their mechanism to overcome productivity shame (guilt feelings if they don’t provide care) best efforts applied, obligation to offer a painless death. Participants also provided emotional and moral support to patients through positive reassurance and comforting talks with patients.

…. You can’t be avoiding the patient because everyone is busy…. So, you have to instead do little more beyond that team…. do the basic things because at least we don’t want that person to go in pain……You don’t want the relatives to be like, these people didn’t really even do anything… (Nurse participant 5).

### Sub theme 3.2: Informants in clinical practice

Student nurses bridge communication gaps between patients and deep connection with relatives. The participants were a source of information for comfort to patients and means to address loneliness and depression of lonely patients. On a negative note; at times when participants were uncomfortable to perform some clinical procedures when overwhelmed by patients’ fragile condition, they preferred to avoid physical presence with dying patients.

“……It’s not easy when patients are reaching the end of their journey, and their condition is deteriorating rapidly. There was a patient who was nearing the end, and some of us felt unease about being present during those last moments. I remember a fellow participant saying, ‘I want to be there for them, but it’s hard to see them like this. I don’t know if I can hold myself together.’“(Nurse Participant 4)

“………. Our role is to provide care and support, but sometimes the reality hits hard. There was a patient who was really struggling, and we needed to perform a procedure to alleviate their pain. One of us expressed, ‘I want to help them, but I’m afraid I’ll make it worse or that I won’t be able to handle their pain. It’s so heart-wrenching to see them like this.’“(Nurse Participant 5)

### Sub theme 3.3: Students’ attitudes to relatives Create Sympathetic relationships with relatives

The participants in their sympathy were protective of their relative’s feelings and hence ensured continuity of care for their sake and not to demoralize them. They tried to be considerate in communication with relatives, consoled and kept them hopeful to minimize the, assumed effects of the patient’s dying process on the family (such as the loss of a person, breadwinner, family breakage, wasted family efforts),

“…. Relatives often felt so lost and overwhelmed, and it was our duty to help guide them through. I recall telling a family, ‘Your loved one’s journey is difficult, but we’re here to make sure you’re not alone in this. Lean on us, and let’s face these challenges together.’“ (Nurse Participant 2)

“……… Communication with relatives had to be gentle and compassionate. I remember a moment when a family was clearly distraught, and I reassured them, ‘We understand the pain you’re going through. Let’s focus on the positive memories and the time you have left to cherish together.’“(Nurse Participant 3)

### Sub-theme 3.4: Cooperate with relatives

The participants tried to keep the relatives informed about the physical process of care and encouraged their participation and involvement in basic physical care such as feeding the dying patient. On the positive note, the participants stated that the relatives were appreciative of the care given when involved.

“…. But these people made sure that everything that was needed was provided…. they supported the decisions emotionally, they bought all medications, they made sure they fed him, even when I would try to feed him, they would help me. ……family members even though they may be sad about their patient by eventually dying, they’ll at least appreciate the care that was offered to the patient….” (Nurse participant 3).

## Discussion

The two themes on perceptions of students identified in this study were described as devastating and frightening reactions that contribute to negative consequences in the care of dying patients, especially during first-time encounters. This could be attributed to the limited clinical, physical, mental psychological, and emotional preparation of the student nurses before exposure to the physical process of care of dying patients. This result is similar to another study that documented that caring for dying patients is a notable stressor for nursing students during clinical practice [18].

In this study, inadequate preparation could have influenced their understanding of the physical process as either hard or torturous experience as expressed by students in this study which later undermines its relevance or positive contribution as a learning opportunity. Studies have documented that there is inadequate provision of end-of-life education to nurses and limited exposure of student nurses to end-of-life care, even though early training could significantly prepare them for their future roles across various nursing units [9].

This concurs with a study done in Sweden that showed registered nurses struggled with understanding of palliative care and transfer of knowledge into clinical practice [19]. As noted in another study Nursing students need to comprehend how to handle a patient’s death, providing compassionate support to the patient’s family, friends, and loved ones as they grapple with death and psychological trauma [11] since they tend to spend more time at the bedside with patients than other healthcare professionals.

However, the exposure of students to the physical process of care is clearly described as important in this study to build confidence and improve knowledge and skills. This is similar to study finding from Australia that showed the relevance of exposure of students to clinical environments and interpersonal encounters that evoke strong emotions. Such environments are good learning strategies and formal support approaches to knowledge development and emotion management which foster professional development and patient care [20].

Exposure of nursing students to care of dying patients therefore could have positive implications on professional progress among health workers which could also be attributed to knowledge exchange and transfer through interactions with senior health workers on wards of dying patients. This finding concurs with a study done in Switzerland in 2020 that showed professional skills improvement among nursing students exposed to caring for terminally ill patients [21].

Additionally, the understanding of the physical process of care and learning gains in such clinical placements seem to be linked to the personal values such as respect and trust. For instance; some participants with positive personal values who feel respected and trusted to care for the dying patient could employ more efforts and lessons learnt than their counterparts who feel un trusted and devalued. A meta-analysis done in 2016 showed that a comfortable and dignified death is important and dependent on the values of nurses in relation to respect for privacy, respect, spiritual peace, and hope [22]. Also, the findings concur with other studies that emphasize the impact of religious beliefs and faith on attitudes toward death and dying patients whereby Faith in God and the afterlife is often associated with a more favorable stance on death and dying, held by nurses, physicians, and the general populace [23].

Therefore, it is important to also consider to understand personal values of nurses and honor them to support nurses during clinical placement and assignments to care for the dying patients.

In this study, there was a notable shift in ideas about the survival of dying patients, the need for end-of-life care and the relevance of the clinical placement. The results are similar to findings in another study that showed even though nursing students often feared dealing with dying patients, concentrated training and palliative care simulations help students shift their attitudes and beliefs and improve the care they give [24].

In this study, the student nurses seemed to come to terms with the reality of death after the clinical placement unlike before when they seemed to live in denial of the fact of death. This result concurs with a study by [25] that recommended combining the students’ academic grasp of palliative care with their practical encounters with death and dying people can be instrumental in assisting the student come to terms with the reality that dying and death is a natural part of life rather than something terrifying.

Findings in this study showed that student nurses recognized and valued the need for end-of-life care after the clinical placement. This concurs with findings in another study done from the Seattle-Tacoma area that showed nurses perceived that patients and families wanted their hopes to be supported at the end of life and often provided this support independent of interactions with physicians [26].

The nursing students in this study finally acknowledged the importance of the clinical placement for their skills improvement. This finding concurs with other studies that have documented consistent satisfaction in clinical placement experiences from student perspectives across semesters and years and significant learning of students in the areas of basic clinical skills, clinical documentation, and collaborative care [27].

Studies have documented that Exposure to positive experiences in the care of end of life in a clinical setting yields expected learning outcomes for undergraduate nursing education while negative emotions decrease the quality of care delivered and increase attrition rates [11].

### Student attitudes towards dying patients

In this study, nursing students displayed a compassionate attitude which steered them to act as informants for dying patients and cooperate with their relatives. In this study, the factors influencing student attitudes were not explored but some studies have shown that educational programs about death and caring for dying patients improved students’ attitude [16] and age, earlier care experience, experiences of meeting a dying person, and place of birth affect students’ attitudes toward the end of life care [28].

In this study, the nursing students seemed to have developed empathic relationships with patients and relatives after frequent interaction with them which could have contributed to their resilience as described by continuation to care even after knowing the outcome of the patient. This is because the nursing students regarded themselves as resourceful persons, who were driven by empathy to relieve the patient of the great pain, and valued the responsibility of care of a patient for the sake of relatives. Empathic relationships between patient and nurse are likely to happen over time due to specific patient needs, nurses’ functions, attributes, and the patient’s reciprocity.

Additionally, the student nurses’ aspiration to provide comfort to end of life and spiritual beliefs could have influenced the empathic relationships displayed in this study. Studies have shown that nurses tend to continue to do their ‘utmost’ work to facilitate a dignified death by focusing on the patient’s comfort and supporting families, despite the complexities and emotional distress they encounter during the end of life care and religious beliefs influence nurses’ commitment and continuation of care[29].

Studies have shown that nurses who develop trusting relationships demonstrate a holistic approach to caring, show their understanding of patients’ suffering, are aware of their unvoiced needs, provide comfort without actually being asked, and are reliable, proficient, competent, and dedicated in their care[30]. Similar to other studies, the nursing students in this study were a source of comforting information to dying patients as a means to address loneliness and depression. Nurses can convey consolation to patients to find peace and reconciliation in the final stages of dying and in so doing may ease some of the existential and spiritual loneliness of dying as they stand with their patients in their suffering.

However, it’s important to note that information delivery may not always be smooth for nursing students, for instance, contrary to this study, a study in the USA showed that students faced challenges in communicating empathically due to dialectic tensions, the burden of carrying bad news, lack of skills for providing empathy, perceived institutional barriers, challenging situations, and perceived dissimilarities between the nurse and the patient delivery[31].

This highlights the need to ensure adequate training and mentoring of nursing students in communication skills so that they play they are able to provide consolation to dying patients and relatives. A study from Uganda in 2014 that showed communication challenges to patients and their relatives, staff shortages, and limited knowledge and resources were perceived as emotionally challenging to nurses and hindrances to their good performance in the provision of palliative care[32].

Studies have shown dying patients’ needs beyond medical treatment rotate around empathy, sympathy, compassion, responsiveness to emotional needs, maintaining hope and a positive attitude, and providing comfort through touch as the emotional support to make them feel unique and special amidst their complex social situation[33].

Similar to other studies the nursing students in this study expressed sympathetic concerns with relatives of dying patients. The participants in their sympathy were protective of relative’s feelings and hence ensured continuity of care for their sake in order not to demoralize them. Sympathy to relatives has been documented as a necessary dimension of care to ease the caregiver’s suffering and ensure compassion and satisfaction with the care provided[34]. Relatives tend to appreciate the sympathy given as emotional support that springs a positive impact to patient and his relatives and this contributes to improved quality of care at end of life.

### Strengths and limitations of the study

This present study was the first of its kind among nursing students and thus provided baseline data.

A limitation of this study pertains to the self-report methodology employed for data collection. The responses provided by participants might have been influenced by social desirability bias, as well as their inclination to portray themselves positively. This potential influence could have implications for the accuracy and depth of the insights conveyed, potentially resulting in a less exhaustive comprehension of the perceptions and experiences under investigation

## Conclusions

There were both positive and negative perceptions of nursing students on caring for dying patients in this study. However, the nursing students seem to develop a shift from negative perceptions at initial information about the process and encounter of caring for a dying patient to positive perceptions during the experience of caring for the dying patient. Students exhibit a compassionate attitude towards dying patients amidst their changing perceptions on caring for dying patients.

### Recommendation

End-of-life care in Uganda still needs more attention and fortunately, there is a massive opportunity for nursing students to participate in the care of dying patients not only as part of skills development or sharpening as learning outcomes for clinical practice but also as enhancement of End-of-life services provision for dying patients who need it but could be missed. Healthcare professionals need to embrace the concept with more positive perceptions to provide quality end-of-life care across all dimensions and categories of patients.

This would require commitment, technical and financial support from the ministry of Health, health facility administrators, and professionals for programs that support and meet both system and patient-centered end-of-life care needs at various health facility levels in the country.

### Future research

There is a need for further investigations into the factors that could influence the emotional and psychological reactions and coping mechanisms among nursing students in the Ugandan context.

## Data Availability

Data sources are available on request. The request can be sent to the corresponding author at ashanabirye1@gmail.com
